# Polyoma virus infection and urothelial carcinoma of the bladder following renal transplantation

**DOI:** 10.1038/sj.bjc.6604711

**Published:** 2008-10-28

**Authors:** I S D Roberts, D Besarani, P Mason, G Turner, P J Friend, R Newton

**Affiliations:** 1 Department of Cellular Pathology, John Radcliffe Hospital, Oxford, UK; 2 Renal Transplant Unit, Churchill Hospital, Oxford, UK; 3 Epidemiology and Genetics Unit, Department of Health Sciences, Hull York Medical School (HYMS) and University of York, UK

**Keywords:** BK virus, bladder cancer, transplant recipients

## Abstract

Renal transplant recipients are at increased risk of bladder carcinoma. The aetiology is unknown but a polyoma virus (PV), BK virus (BKV), may play a role; urinary reactivation of this virus is common post-renal transplantation and PV large T-antigen (T-Ag) has transforming activity. In this study, we investigate the potential role of BKV in post-transplant urothelial carcinoma by immunostaining tumour tissue for PV T-Ag. There was no positivity for PV T-Ag in urothelial carcinomas from 20 non-transplant patients. Since 1990, 10 transplant recipients in our unit have developed urothelial carcinoma, and tumour tissue was available in eight recipients. Two patients were transplanted since the first case of PV nephropathy (PVN) was diagnosed in our unit in 2000 and both showed PV reactivation post-transplantation. In one of these patients, there was strong nuclear staining for PV T-Ag in tumour cells, with no staining of non-neoplastic urothelium. We conclude that PV infection is not associated with urothelial carcinoma in non-transplant patients, and is uncommon in transplant-associated tumours. Its presence in all tumour cells in one patient transplanted in the PVN era might suggest a possible role in tumorigenesis in that case.

Renal transplant recipients carry a three- to four-fold increased risk of urothelial carcinoma when compared with the general population, the cause of which is unknown (Birkeland *et al*, 1995; Buzzeo *et al*, 1997; Vajdic *et al*, 2006). Infection with oncogenic viruses is one possible explanation, and BK virus (BKV) is a potential candidate virus.

BKV, a polyoma virus (PV), was first identified in 1971, in a renal transplant recipient with ureteric stenosis (Gardner *et al*, 1971). It infects 90% of the population during childhood and subsequently remains latent in the epithelium of the urinary tract. Urinary reactivation of the virus is seen in approximately 30% of renal transplant recipients receiving modern immunosuppressive protocols (Thamboo *et al*, 2007). Persistent high levels of urothelial reactivation are associated with an increased risk of infection of the renal tubules within the graft, resulting in PV nephropathy (PVN) that occurs in 3–5% of renal transplant recipients. This is a relatively recent phenomenon that was first described in 1995 (Purighalla *et al*, 1995). The first patient with PVN in our unit in Oxford was diagnosed in 2000 (Thamboo *et al*, 2007). Current potent immunosuppressive regimens, the combination of tacrolimus and mycophenolate mofetil (MMF) in particular, have been implicated in the emergence of this complication.

The polyoma viruses, including BKV, have transforming activity, although a role in human neoplasia is yet to be demonstrated. PV expresses a viral oncogene, the large T-antigen (T-Ag) that inactivates the pocket protein family, including pRb. It has recently been demonstrated that the BKV T-Ag activates the DNA methyltransferase 1 gene – DNMT1 (McCabe *et al*, 2006). DNMT1 is associated with tumorigenesis through tumour suppressor gene hypermethylation.

In this study, we investigate the potential role of BKV in urothelial carcinoma in immunocompetent individuals and following renal transplantation by staining of tumour tissue with antibody against PV T-Ag.

## Materials and methods

### Patients

Search of the local renal transplant database (1990–2007) revealed 10 patients with post-transplant urothelial carcinoma of the bladder in our unit, eight of whom had archival paraffin blocks of tumour tissue available for study. A control group of immunocompetent individuals with carcinoma of the bladder was identified. These were 20 consecutive non-transplant patients diagnosed with high-grade invasive urothelial carcinoma in 2006.

Ethics approval was obtained for the study of bladder tumours and renal tissue from renal transplant recipients (ethics committee reference numbers O-02.062 and 04/Q1606/96).

### Immunohistochemistry (IH)

Immunohistochemistry for PV T-Ag was performed using a primary monoclonal anti-SV40 T-Ag (Calbiochem, San Diego, CA, USA). This antibody stains all human polyoma viruses, including BKV. Before incubation with the antibody, antigen retrieval was performed using a pressure cooker for 60–90 s at high pressure, with paraffin sections in 0.01 M citrate buffer at pH 6. Primary antibody staining was done for 30 min at a dilution of 1/200. The detection system used was Vector Elite ABC, Burlingame, CA, USA.

## Results

The 20 non-transplant tumours were all negative for PV T-Ag on IH.

The patients with transplant-associated urothelial carcinoma are summarised in Table 1. Of the eight patients with tissue available for review and staining, the mean age at diagnosis was 58 years and male/female ratio was 2 : 6. Two patients (7 and 8) were transplanted since the first case of BKV nephropathy was diagnosed in Oxford in 2000. Both patients showed post-transplant reactivation of BKV, one diagnosed on renal biopsy and the other on urine cytology.

**Figure 1:**
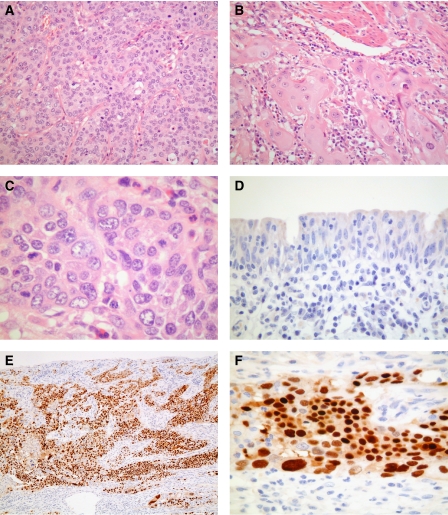
Tumour tissue from patient 7, showing high-grade invasive urothelial carcinoma (**A**), with focal squamous differentiation (**B**). High power (**C**) reveals no evidence of typical viral inclusions on H&E stain. Immunohistochemistry for PV T-Ag is negative in non-neoplastic urothelium adjacent to the tumour (**D**), but shows intense nuclear staining in almost all tumour cells (**E** and **F**).

One of eight urothelial carcinomas in transplant recipients (patient 7) showed strong nuclear staining for PV T-Ag in virtually all tumour cells, with negative staining in adjacent non-neoplastic urothelium (Figure 1). Typical intranuclear inclusions were not seen on H&E sections and there was no lymphocytic response to the PV-positive tumour cells, even following reduced immunosuppression for 6 weeks after diagnosis and before cystectomy.

**Figure 2:**
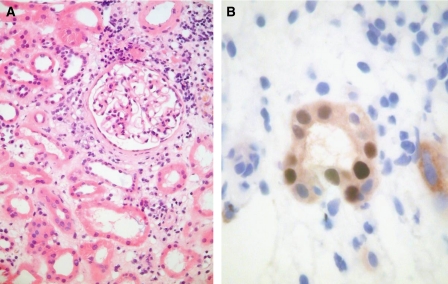
Renal biopsy 2 years post-transplantation from patient 7, showing a minor non-specific inflammatory cell infiltrate on H&E (**A**). Immunohistochemistry for PV T-Ag (**B**) shows staining of tubular epithelial cell nuclei, confirming a diagnosis of PVN.

Patient 7 is a 40-year-old woman, who received a renal transplant in 2001. Her initial immunosuppression was ciclosporin, azathioprine and prednisolone. However, she suffered three early rejections: a Banff 97 type IA rejection at day 7 post-transplantation, treated with 3 days of pulse methylprednisolone; a Banff 97 type III rejection at 13 days post-transplantation, treated with antithymocyte globulin and a Banff 97 type IB rejection, diagnosed at 27 days post-transplantation. The latter was treated with a second 3-day course of pulse methylprednisolone, followed by switch of ciclosporin and azathioprine to tacrolimus and MMF. No urine cytology to detect PV reactivation was performed, but PVN was diagnosed retrospectively using IH for PV T-Ag in a renal biopsy performed for graft dysfunction at 2 years post-transplantation (Figure 2). This showed a minor non-specific infiltrate with no viral inclusions apparent on the H&E stain. Her bladder tumour was diagnosed 4 years post-transplantation and was treated with radical cystectomy, bilateral native nephrectomy and hysterectomy. Her renal allograft shows good functioning 6 years post-transplantation (serum creatinine 137 *μ*mol l^−1^).

**Figure 3:**
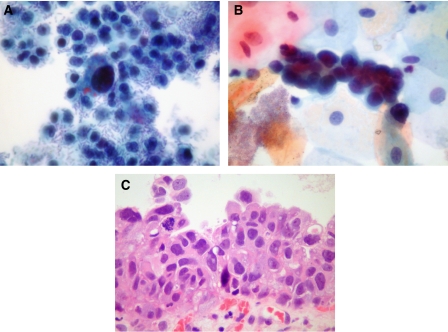
Urine cytology in patient 8 initially suggested BKV infection (**A**) but a sample 3 months later was more in keeping with urothelial carcinoma (**B**), confirmed as carcinoma *in situ* on biopsy (**C**).

Patient 8 is a 65-year-old woman, transplanted in 2004. She developed BKV reactivation 5 months post-transplantation, diagnosed on urine cytology, but did not have biopsy-proven BKV nephropathy. She developed graft dysfunction 2.5 years post-transplantation, biopsy showing chronic damage with active rejection. Urine cytology at this time showed large numbers of atypical cells distributed singly, which were initially interpreted as decoy cells, indicative of BKV infection (Figure 3A). However, in the third urine specimen (3 months after the first), aggregates of atypical cells were present, suggesting high-grade urothelial carcinoma (Figure 3B). Urothelial carcinoma *in situ* was confirmed on biopsy (Figure 3C) and this was negative for PV T-Ag.

## Discussion

There is conflicting evidence of a role for BKV in urothelial carcinoma in immunocompetent individuals. Weinreb *et al* (2006) reported a significant association between urine cytology suggestive of PV infection and subsequent diagnosis of bladder carcinoma. However, this study did not confirm PV positivity with IH and the urinary findings may have been secondary to early urothelial carcinoma. The distinction of BKV infection from urothelial malignancy can be very difficult on cytomorphology alone, as illustrated by a recent study (Glatz *et al*, 2006) and by case 8 in our series. In a case–control study, Newton *et al* (2005) found no association between prevalence or titres of anti-BKV antibodies and diagnosis of bladder cancer. A recent tissue-based study detected BKV DNA by PCR in only 5.5% of urothelial carcinomas, all of which were negative on IH for BKV T-Ag (Rollison *et al*, 2007). Similarly, we found no evidence of PV T-Ag in urothelial carcinoma from 20 immunocompetent patients.

There has been no previous systematic study of BKV in transplant-associated urothelial carcinoma. Geetha *et al* (2002) reported a single patient who developed PVN following simultaneous pancreas–kidney transplant and who subsequently developed carcinoma of the bladder that was diffusely positive for PV T-Ag on IH, confirmed to be BKV by PCR. Patient 7 in our series closely resembles this case. PV T-Ag positivity in the tumour tissue of our patient implicates a polyoma virus in tumorigenesis but is not specific for BKV. Although this is the most likely candidate virus, BKV-specific PCR was not performed and the presence of another polyoma virus could not be excluded. The association of PV-positive urothelial carcinoma and PVN in these patients suggests that persistent or high levels of post-transplant urinary reactivation of BKV may have a pathogenic role in the development of urothelial carcinoma. Alternatively, it is possible that tumour cells are more susceptible to BKV infection than normal urothelium, as infection occurs primarily in proliferating cells, and that positivity is a consequence rather than cause of neoplastic transformation. Urine cytology screening for BKV reactivation post-transplantation is now routine in many units, including ours. Long-term follow-up of these patients is required to demonstrate an association between viral reactivation and subsequent tumour development, and thus confirm the potential oncogenic role of BKV.

## Figures and Tables

**Table 1:** Patients with post-renal transplant urothelial carcinoma (UC) of the bladder

**Patient number**	**Sex/age at diagnosis**	**Transplant year**	**Time from transplant to UC diagnosis (months)**	**Grade/stage of UC at diagnosis**	**PV T-Ag staining**
1	F/75 years	1990	129	Low grade/Ta	Negative
2	M/53 years	1990	38	High grade/T1	Negative
3	F/69 years	1992	121	High grade/T2	Negative
4	F/41 years	1993	147	High grade/T2	Negative
5	F/56 years	1995	68	High grade/T2	Negative
6	M/61 years	1996	129	High grade/T3	Negative
7	F/40 years	2001	54	High grade/T3	Positive
8	F/65 years	2004	33	High grade/Tis	Negative
